# Prevalence of and factors related to anemia among Japanese adult women: Secondary data analysis using health check-up database

**DOI:** 10.1038/s41598-019-52798-y

**Published:** 2019-11-19

**Authors:** Kanako Hisa, Megumi Haruna, Naoko Hikita, Emi Sasagawa, Kaori Yonezawa, Maiko Suto, Erika Ota

**Affiliations:** 1 Department of Midwifery and Women’s Health, Division of Health Science and Nursing, Graduate School of Medicine, The University of Tokyo, Tokyo, Japan; 2 Department of Health Policy, National Center for Child Health and Development, Tokyo, Japan; 3 Global Health Nursing, Graduate School of Nursing Science, St. Luke’s International University, Tokyo, Japan

**Keywords:** Epidemiology, Risk factors

## Abstract

The issue of anemia is important in terms of a woman’s preconception health. This study aimed to conduct an exploratory investigation of the prevalence of and factors related to anemia in non-pregnant Japanese women. Secondary data analysis was conducted using a database of women aged 20–49 years old who had attended an annual health check-up at a hospital in Tokyo (n = 10,598). A multiple logistic regression analysis was performed to identify factors related to anemia in two age groups: women aged 34 and under and those aged 35 and over. Anemia was defined as hemoglobin concentration levels <12.0 g/dL. The overall proportion of women with Hb <12.0 g/dL was 17.1%. Women aged 35 and over with a current medical history of uterine myoma were found to be at a higher risk of anemia. Women aged 35 and over who were overweight had a lower risk of anemia than women with normal weights. Current and past smoking habits affected hemoglobin levels among women aged 35 and over. In both age groups, those who drank alcohol habitually were at a lower risk than those who did not. Related factors of anemia should take into consideration a woman’s age and lifestyle.

## Introduction

Anemia is a common health and nutritional problem worldwide. Women are especially prone to anemia because they menstruate and experience pregnancy and childbirth. The World Health Organization (WHO) defines anemia as ‘a condition in which the number of red blood cells (and consequently, their oxygen-carrying capacity) is insufficient to meet the body’s physiologic needs’^1^. Recognized symptoms include shortness of breath, palpitations, malaise, headache, and dizziness. In addition, anemia has been found to cause reduced performance in cognitive attention and reduced working ability^2,3^.

Globally, regions with a high prevalence of anemia have populations suffering at higher rates from malnutrition and infectious diseases due to unsanitary living conditions, as found in developing countries. However, the prevalence of anemia in Japanese women of reproductive age is 22%, which is high compared to other developed countries^4^. The prevalence of anemia among non-pregnant women aged 15–49 years old is reported to be 12% in United States of America and 17% in Australia^4^. In addition, 31% of Japanese women suffer from anemia during pregnancy^4^. A prior study has shown that a significantly higher risk of low birth weight and preterm birth (with adjusted odds ratios of 1.29 and 1.21, respectively) is associated with anemia during early or mid-term pregnancy^5^.

Measures to prevent anemia are recommended, when a woman is planning a pregnancy or becomes pregnant unexpectedly. In recent years the WHO and the Centers for Disease Control and Prevention in the USA have clarified the importance of preconception health^6,7^. This concept is related to the notion that improving the overall health of all women of reproductive age has a positive impact on the health of mothers and children after pregnancy^6,7^. There are several reasons for which it is desirable to prevent anemia before pregnancy in adult women. First, it takes 2–3 months to improve levels of stored iron, which is the fundamental problem underlying the iron deficiency anemia^8^. Pregnant women with a tendency to be anemia may be at an increased risk of the condition during weeks 5–10 of gestation, the critical period of fetal organ development. Second, according to previous research, the nutritional intake of Japanese women during pregnancy is almost the same as before pregnancy despite the body’s increased demand for iron during pregnancy, which is lower than the nutritional intake recommended by the Ministry of Health, Labour and Welfare^9,10^. In addition, for women who have morning sickness, it can be difficult to take iron supplements during the early stage of pregnancy^11^.

Pregnancy at advanced maternal age is increasingly common in Japan. The proportion of births in which the maternal age is 35 years or older has drastically increased in Japan from 9.5% in 1995 to 16.3% in 2005 and 28.9% in 2017^12^. Additionally, previous studies have revealed that pregnant women aged 35 and over tend to be more likely to have anemia than those aged 20–34 years^13^. However, the reason for this is not explicitly explained. If the factors related to anemia in women differ according to age, it is possible that adequate measured for preventing anemia corresponding to these differences are not currently being undertaken by women and their healthcare providers. In fact, it has been pointed out that women in their late 30 s, and especially in their 40 s tend to suffer from anemia that can be attributed to increased menorrhagia associated with gynecological diseases and irregular genital bleeding with unknown causes^8^. However, it is not clear whether factors other than gynecological diseases related to anemia, such as lifestyle habits, or other related factors of anemia differ according to a woman’s age.

If the factors associated with anemia in terms of age are clarified, interventions can be developed to prevent anemia in adult women in Japan. The aims of this study were to investigate the prevalence of and factors related to anemia among non-pregnant Japanese women by age group- women aged 34 and under, and women 35 and over.

## Results

### Participants’ characteristics and prevalence of anemia

**Figure 1:**
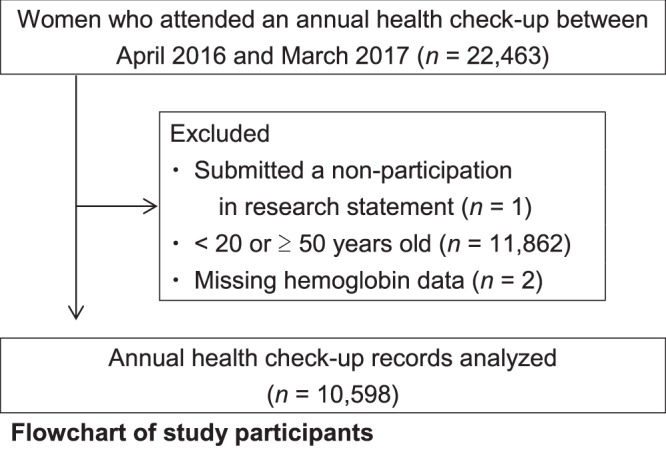
Flowchart of study participants.

After excluding one woman who had submitted a non-participation in research statement, the 11,862 who fell outside the age range of 20–49 years, and two who were with missing hemoglobin (Hb) data, the remaining data set consisted of 10,598 women’s annual health check-up records (Fig. 1). The median age of participants was 42.0 years. There were no participants with outlying Hb values.

Table 1 shows the hematological data. There were 1,811 (17.1%) women with Hb <12.0 g/dL (263 women [13.9%] aged 34 and under and 1,548 [17.8%] aged 35 and over). There were 1,162 (11.0%), 626 (5.9%), and 23 (0.2%) women with mild, moderate and severe anemia, respectively. More than 10% of all age groups had not self-reported a current medical history of anemia in the questionnaire, despite having Hb <12.0 g/dL.

**Table 1:** Laboratory data and prevalence of anemia among Japanese adult women (n = 10,598).

	All	20–24 years (n = 312)	25–29 years (n = 604)	30–34 years (n = 973)	35–39 years (n = 1,901)	40–44 years (n = 2,954)	45–49 years (n = 3,854)
	N(%) or Mean ± SD						
Hemoglobin (g/dL)	12.82 ± 1.14	12.89 ± 0.92	12.83 ± 1.07	12.87 ± 1.00	12.85 ± 1.04	12.76 ± 1.16	12.82 ± 1.23
<12 g/dL	1,811 (17.1)	45 (14.4)	85 (14.1)	133 (13.7)	284 (14.9)	551 (18.7)	713 (18.5)
Type of anemia							
Mild	1,162 (11.0)	34 (10.9)	61 (10.1)	93 (9.6)	196 (10.3)	351 (11.9)	427 (11.1)
Moderate	626 (5.9)	11 (3.5)	22 (3.6)	39 (4.0)	87 (4.6)	192 (6.5)	275 (7.1)
Severe	23 (0.2)	0	2 (0.3)	1 (0.1)	1 (0.1)	8 (0.3)	11 (0.3)
Current medical history of anemia	184 (1.7)	1 (0.3)	4 (0.7)	5 (0.5)	18 (0.9)	51 (1.7)	105 (2.7)
Women with Hb < 12 g/dL and who had not self-reported a current medical history of anemia	1,721 (16.2)	45 (14.4)	83 (13.7)	131 (13.5)	278 (14.6)	523 (17.7)	661 (17.2)

Mild anemia: Hb 11.0 to 11.9 g/dL; moderate anemia: Hb 8.0 to 10.9 g/dL; severe anemia: Hb < 8.0 g/dL.

### Factors associated with anemia in japanese women

Table 2 shows participants’ characteristics and comparisons between women with and without anemia using univariate analysis. Body fat percentage and drinking habits differed significantly between these groups both among women aged 34 and under, and among those aged 35 and over. In women aged 35 and over, there were also significant differences between the groups in age, weight change over the past year, uterine myoma, duodenal ulcer, smoking habits, number of days per week with alcohol consumption.

**Table 2:** Participants’ socio-demographic characteristics (n = 10,598) .

	20–34 years (n = 1,889)			*p value*	35–49 years (n = 8,709)			*p value*
	All	Anemia	Without anemia		All	Anemia	Without anemia	
		n = 263	n = 1,626			n = 1,548	n = 7,161	
		13.9%	86.1%			17.8%	82.2%	
	Median (IQR^a^) or N (%) or Mean ± SD				Median (IQR^a^) or N (%) or Mean ± SD			
**Age (years)**	30.0 (26.0–32.0)	30.0 (26.0–32.0)	30.0 (26.0–32.0)	0.729^b^	44.0 (40.0–47.0)	44.0 (41.0–47.0)	44.0 (40.0–47.0)	**0.008**^**b**^
**Body mass index (kg/m**^2^**)**				0.445^c^				0.051^c^
less than 18.5	402 (21.3)	48 (18.3)	354 (21.7)		1,503 (17.3)	262 (16.9)	1,241 (17.3)	
18.5–24.9	1,375 (72.8)	201 (76.4)	1,174 (72.2)		6,308 (72.4)	1,154 (74.5)	5,154 (72.0)	
25.0–29.9	96 (5.1)	13 (4.9)	83 (5.1)		720 (8.3)	102 (6.6)	618 (8.6)	
30.0 or more	16 (0.8)	1 (0.4)	15 (0.9)		177 (2.0)	30 (1.9)	147 (2.1)	
**Weight change over the past year**				0.067^c^				**0.021**^**c**^
No change	1,384 (73.3)	208 (79.1)	1,176 (72.3)		6,532 (75.0)	1,203 (77.7)	5,329 (74.4)	
3 kg or more increase	343 (18.2)	39 (14.8)	304 (18.6)		1,750 (20.1)	273 (17.6)	1,477 (20.6)	
3 kg or more decrease	161 (8.5)	16 (6.0)	145 (8.4)		427 (4.9)	72 (4.7)	355 (5.0)	
**Current medical history**								
Adenomyosis	8 (0.4)	0 (0.0)	8 (0.5)	0.301^d^	127 (1.5)	30 (1.9)	97 (1.4)	0.082^c^
Endometriosis enterna (e.g. chocolate cyst, endometriosis cyst)	30 (1.6)	2 (0.8)	28 (1.7)	0.190^d^	241 (2.8)	40 (2.6)	201 (2.8)	0.628^c^
Uterine myoma	20 (1.1)	4 (1.5)	16 (1.0)	0.300^d^	827 (3.5)	202 (13.0)	625 (8.7)	**<0.001**^**c**^
Cervical cancer	2 (0.1)	1 (0.4)	1 (0.1)	0.259^d^	48 (0.6)	7 (0.5)	41 (0.6)	0.562^c^
Endometrial cancer	1 (0.1)	0 (0.0)	1 (0.1)	0.861^d^	13 (0.1)	1 (0.1)	12 (0.2)	0.299^d^
Ovarian tumor (malignant/benign)	10 (0.5)	2 (0.8)	8 (0.5)	0.416^d^	122 (1.4)	20 (1.3)	102 (1.4)	0.688^c^
Abnormality of uterine cytology	35 (1.9)	5 (1.9)	30 (1.8)	0.550^d^	315 (3.6)	47 (3.0)	268 (3.7)	0.177^c^
Breast cancer/mastopathy	9 (0.5)	1 (0.4)	8 (0.5)	0.637^d^	374 (4.3)	66 (4.3)	308 (4.3)	0.947^c^
Gastric ulcer	0	0	0		5 (0.1)	2 (0.1)	3 (0.0)	0.218^d^
Duodenal ulcer	0	0	0		5 (0.1)	3 (0.2)	2 (0.0)	**0.042**^**d**^
Ulcerative colitis	3 (0.2)	1 (0.4)	2 (0.1)	0.362^d^	25 (0.3)	7 (0.5)	18 (0.3)	0.141^d^
**Body fat percentage**	24.8 ± 5.6	24.1 ± 5.2	25.0 ± 5.6	**0.026**^**e**^	25.8 ± 6.33	24.8 ± 5.8	26.0 ± 6.4	**<0.001 **^**f**^
**Smoking habits**				0.245^c^				**<0.001**^**c**^
Non-smoker	1,689 (89.5)	243 (92.3)	1,446 (88.9)		7,110 (81.6)	1,340 (86.6)	5,770 (80.6)	
Current smoker	47 (2.5)	5 (1.9)	42 (2.6)		422 (4.8)	58 (3.7)	364 (5.1)	
Smoked in the past	152 (8.1)	15 (5.7)	137 (8.4)		1,177 (13.5)	150 (9.7)	1,027 (14.3)	
**Number of tobacco per day**	8.0 (5.0–12.0)	8.0 (5.0–10.0)	8.0 (4.0–12.0)	0.825^b^	10.0 (5.0–15.0)	10.0 (5.0–10.0)	10.0 (5.0–15.0)	0.119^b^
**Drinking habits**				**0.015**^**c**^				**<0.001**^**c**^
Non-drinker	1,058 (56.0)	169 (64.3)	889 (54.7)		4,195 (48.2)	813 (52.5)	3,382 (47.2)	
Occasional drinker	306 (16.2)	36 (13.6)	270 (16.6)		1,464 (16.8)	289 (18.7)	1,175 (16.4)	
Habitual drinker	524 (27.8)	58 (22.1)	466 (28.7)		3,050 (35.0)	446 (28.8)	2,604 (36.4)	
**Number of days per week with alcohol consumption**	2.0 (1.0–4.0)	2.0 (1.0–3.25)	2.0 (1.0–4.0)	0.149^b^	3.0 (1.0–5.0)	2.0 (1.0–5.0)	3.0 (1.0–5.0)	**0.001**^**b**^
**Sleeping habits**				0.152^c^				0.072^c^
7 hours or more	299 (29.3)	36 (13.6)	263 (16.2)		1,273 (24.5)	205 (22.2)	1,068 (25.0)	
6 hours or below	722 (70.7)	112 (42.5)	610 (37.5)		3,913 (75.5)	717 (77.8)	3,196 (75.0)	
**Exercise habits** (light exercise, including walking for more than 20 minutes)				0.892^c^				0.284^c^
No exercise	837 (44.3)	113 (42.9)	724 (44.5)		3,509 (40.3)	655 (42.3)	2,854 (39.9)	
Exercising 1–2 days per week	677 (35.9)	97 (36.8)	580 (35.7)		3,250 (37.3)	557 (36.0)	2,693 (37.6)	
Exercising 3–5 days per week	240 (12.7)	36 (13.6)	204 (12.5)		1,370 (15.7)	242 (15.6)	1,128 (15.8)	
Exercising almost every day	134 (7.1)	17 (6.4)	117 (7.2)		580 (6.7)	94 (6.1)	486 (6.8)	

a: IQR = interquartile range (25th – 75th percentile), b: Mann-Whitney U test, c: Chi-square test, d: Fisher’s exact test, e: Student’s *t*-test, f: Welch’s *t*-test.

Anemia: Hb < 12.0 g/dL

Without anemia: Hb ≥ 12.0 g/dL

20–34 years: n = 1,888 for Weight change over the past year, Smoking habits, Drinking habits, Exercise habits. n = 1,886 for Body fat percentage. n = 199 for Number of tobacco per day. n = 830 for Number of days per week with alcohol consumption. n = 1,021 for Sleeping habits.

35–49 years: n = 8,708 for Body Mass Index. n = 8,706 for Body fat percentage. n = 1,599 for Number of tobacco per day. n = 4,514 for Number of days per week with alcohol consumption. n = 5,186 for Sleeping habits.

Table 3 shows the results of a multiple logistic regression analysis. In the group aged 34 and under, the correlation coefficient between body mass index (BMI) and body fat percentage was 0.91. Therefore, only BMI was used for the analysis. In the group aged 35 and over, the correlation coefficients between BMI and body fat percentage and between drinking habits and number of days of alcohol consumption per week were 0.93 and 0.65, respectively. Therefore, only BMI and drinking habits were used for the analysis. Women who were aged 34 and under and also reported drinking alcohol habitually had a lower odds ratio of having anemia than women in that age group who reported that they did not drink alcohol (adjusted odds ratio [AOR] = 0.67, 95% confidence interval [CI]: 0.48–0.92). Among the sample of women aged 35 and over, women who self-reported a current medical history of uterine myoma had higher odds of having anemia (AOR = 1.53, 95% CI: 1.29–1.82) than women aged 35 and over who did not report this medical history. Among the sample of women aged 35 and over, who were overweight, there were lower odds of anemia than in women who were of normal weights (AOR = 0.74, 95% CI: 0.59–0.92). Based on an analysis of the self-reports on this variable, there were lower odds of anemia among women aged 35 and over who reported a weight gain of 3 kg or more in the past year compared to women aged 35 and over who had not undergone weight change (AOR = 0.85, 95% CI: 0.73–0.99).; among women who reported smoking currently and having smoked in the past compared to those who reported not having smoked (AOR = 0.75, 95% CI: 0.56–0.99; and AOR = 0.67, 95% CI: 0.56–0.81); and among women who reported drinking alcohol habitually compared to those who reported not drinking alcohol (AOR = 0.73, 95% CI: 0.64–0.83).

**Table 3:** Effects of factors related to anemia as indicated by multiple logistic regression (n = 7,074).

Variables	20–34 years (n = 1,888)								35–49 years (n = 5,186)							
	Unadjusted Odds Ratio (OR)	95%CI^a^		*p value*	Adjusted Odds Ratio (AOR)	95%CI^a^		*p value*	Unadjusted Odds Ratio (OR)	95%CI^a^		*p value*	Adjusted Odds Ratio (AOR)	95%CI^a^		*p value*
		lower	upper			lower	upper			lower	upper			lower	upper	
**Age (years)**	0.99	0.96	1.03	0.706	1.01	0.97	1.04	0.732	1.02	1.00	1.03	**0.031**	1.01	1.00	1.03	0.131
**Body mass index (kg/m**^**2**^**)**																
less than 18.5	0.79	0.57	1.11	0.174	0.78	0.56	1.09	0.147	0.94	0.81	1.09	0.435	0.93	0.80	1.08	0.314
18.5–24.9	Reference				Reference				Reference				Reference			
25.0–29.9	0.92	0.50	1.67	0.772	0.91	0.50	1.68	0.772	0.74	0.59	0.92	**0.006**	0.74	0.59	0.92	**0.007**
30.0 or more	0.39	0.05	2.96	0.362	0.37	0.05	2.83	0.337	0.91	0.61	1.36	0.648	0.92	0.61	1.38	0.685
**Weight change over the past year**																
No change									Reference				Reference			
3 kg or more increase									0.82	0.71	0.95	**0.006**	0.85	0.73	0.99	**0.034**
3 kg or more decrease									0.90	0.69	1.17	0.421	0.91	0.70	1.19	0.488
**Current medical history**																
Uterine myoma									1.57	1.33	1.86	**<0.001**	1.53	1.29	1.82	**<0.001**
Duodenal ulcer									6.95	1.16	41.6	**0.034**	5.71	0.93	35.2	0.060
**Smoking habits**																
Non-smoker	Reference				Reference				Reference				Reference			
Current smoker	0.71	0.28	1.81	0.471	0.77	0.30	1.98	0.586	0.69	0.52	0.91	**0.009**	0.75	0.56	0.99	**0.045**
Smoked in the past	0.65	0.38	1.13	0.127	0.69	0.39	1.21	0.191	0.63	0.53	0.75	**<0.001**	0.67	0.56	0.81	**<0.001**
**Drinking habits**																
Non-drinker	Reference				Reference				Reference				Reference			
Occasional drinker	0.70	0.48	1.03	0.071	0.70	0.48	1.03	0.070	1.02	0.88	1.19	0.764	1.02	0.88	1.18	0.824
Habitual drinker	0.66	0.48	0.90	**0.009**	0.67	0.48	0.92	**0.015**	0.71	0.63	0.81	**<0.001**	0.73	0.64	0.83	**<0.001**

Anemia (Hb < 12.0 g/dL); without anemia (Hb ≥ 12.0 g/dL).

Convariates: 20–34 years; Age, Body mass index, Smoking habits, Drinking habits.

Convariates: 35–49 years; Age, Body mass index, Weight change over the past year, Uterine myoma, Duodenal ulcer, Smoking habits, Drinking habits.

a: CI = confidence interval.

When 0.3 g/dL was subtracted from the measured Hb of those women who reported in the questionnaire that they were current smokers, the number of women with anemia increased from 1,811 to 1,835; however, the results of the analysis did not change (data not shown).

## Discussion

This study investigated the prevalence of and factors related to anemia among Japanese adult women. In women aged 35 and over, the risk of anemia was found to be higher in those with a current medical history of uterine myoma; in contrast, being overweight, having undergone a weight gain of 3 kg or more the past year, currently smoking, and having a history of smoking were associated with a lower risk of anemia. In both age groups, the women who drank alcohol habitually were at a lower risk than those women who reported not drinking.

In this study, the percentage of Japanese women aged 20–49 with Hb <12.0 g/dL was 17.1%. According to the WHO, 22% of Japanese non-pregnant women aged 15–49 were anemic^4^, and the 2015 National Health and Nutrition Survey in Japan reported that the proportion of women with Hb <12.0 g/dL was 16.7% among those aged 20–29 years, 23.1% among those aged 30–39, and 20.8% among those aged 40–49 (excluding those who were taking medications as treatment for anemia^14^). Compared to the national data, the prevalence of anemia in the participants in this study, who had received an annual health check-up at one hospital in Tokyo, tended to be lower. The high proportion of women who did not self-report a current medical history of anemia despite having Hb indicative of anemia, thus highlights the importance of an annual health check-up that includes a blood test for Hb.

In women aged 35 and over, those who had a current medical history of uterine myoma were more likely to become anemic. An association between gynecological diseases and abnormal genital bleeding (including heavy menstrual bleeding) has been pointed out. Previous studies have noted that heavy menstrual bleeding caused by gynecological diseases (such as leiomyoma, adenomyosis, abnormality of the endometrium, malignancy, and ovulation disorders) is particularly common in women since their late 30 s, especially in their 40 s^8,15^. It is reasonable to assume that the association between gynecological diseases and anemia for women aged 35 and over in this study was clear. The gynecological diseases that most related to anemia in this study was uterine myoma. Uterine myoma is a condition in which benign tumors arising from the smooth muscle of the uterine body^15^, and a estrogen-dependent diseases. In the modern era, many women experience a large number of menstruation cycles in their lifetimes, as a result of a combination of factors: earlier menarche, a declining birth-rate, and later menopause^16^. An increase in the number of menstruation cycles implies that women are exposed to estrogen longer, and the risk of developing estrogen-dependent diseases may, therefore increase. In this study, there was no significant association between specific gynecological diseases and anemia in women aged 34 and under, which could be because women aged 34 and under have not yet been diagnosed with gynecological diseases owing to the lack of serious symptoms. For women aged 35 and over who are planning a pregnancy and have symptoms of anemia or abnormal genital bleeding (including heavy menstrual bleeding), a regular check-up for early detection of gynecological diseases in terms of preconception health is recommended.

Women aged 35 and over and overweight had a lower risk of anemia compared to women of normal weights. Previous studies have shown an association between BMI and anemia; however, the direction of association varies. According to one study, adolescents who are overweight or obese are prone to iron deficiency anemia because iron requirements are higher as a function of weight and iron-poor food^17^. On the other hand, one study of non-pregnant ever-married women pointed out that undernourished women were shown to have a lower Hb and more likely to become anemic than normal, overweight, and obese women^18^. A study of pregnancy anemia showed that women with an underweight pre-pregnancy BMI are more likely to become anemic after pregnancy than women with a normal weight, and women with an overweight or obese pre-pregnancy BMI are less likely to develop anemia after pregnancy^19^. The reason that being underweight is associated with anemia can be explained by the lack of nutrition that affects anemia. The results of this study support this direction of association. In addition, these results show a lower risk of anemia among women who are aged 35 and over and have reported a weight gain of 3 kg or more in the previous year compared to those women who reported no weight change. The relationship between weight change and anemia was first revealed in this study; however, the causal inference had not been found. Weight change over a year may be affected by dietary habits. In addition, for women aged 35 and over, various factors such as, decreasing basal metabolic rate^20^, change in hormonal balance, and the influence of inappropriate lifestyles long-term may affect weight change.

For both age groups examined in this study, the risk of anemia was lower among women who reported drinking alcohol habitually than women who reported not drinking. The direct causal relationship for this association could not be explained clearly. The possibility of reversed causality is conceivable; that is women without anemia might be active enough to able to drink alcohol habitually. Regardless, the results of this study do not imply that alcohol use is a method of reducing the risk of anemia. There may also be a third variable, such as diet that reads to this association. The reason for the association between habitual drinking and the reduced risk of anemia observed in this study may be that the women’s dietary and nutritional intake can vary depending on their drinking habits. In many developed countries, anemia is associated with dietary intake, and such factors may pertain to deficiencies of micronutrients including iron, iodine, and vitamin B_12_, as well as a lower intake of meat, fruit, and vegetables^2,21^. As accurate data on the participants’ dietary intake were not available in this study, future studies should investigate the role of dietary intake along with drinking habits.

This study found that women aged 35 and over who reported currently smoking and having smoked in the past were at relatively low risk of anemia compared to those who reported not smoking. In terms of the physiological mechanism that explains why smoking increases Hb, carbon monoxide (CO) binds with Hb in the blood to produce carboxyhemoglobin, which is abnormal Hb without the ability to transport oxygen. This increases the production of erythrocytes, which in turn, increases the apparent Hb^22^. The larger the amount of tobacco smoked and the longer the duration of an individual’s smoking habit, the greater the rise in average Hb^23^. As this mechanism has already been established, an observed association between current smoking and anemia was anticipated. In addition, past smoking history was also associated with anemia in this study. This might be because the women underreported their smoking habits in the self-report question. The results of this study should not be interpreted as indicating that smoking decreases the risk of anemia; instead, women’s smoking habits, both currently and in the past, affect their Hb values.

This study has several limitations. First, the cross-sectional nature of this study did not allow for causal inference; therefore, there was no indication of the direction of the relationship between anemia, weight change, drinking habits, and smoking habits. Second, because all data were obtained from a single hospital, the results might not be generalizable to all Japanese women. Third, self-reported current medical history and lifestyle information were used based on the participants’ questionnaire responses. It is possible that some participants did not recall the correct names for certain aspects of their current medical history. However, one benefit of using health check-up data is that there is little laboratory bias in blood test results because the same tests are conducted for every participant. Fourth, accurate data on the participants’ nutritional intake and gynecology-specific data could not be obtained from their health check-up data. Many previous studies have demonstrated how eating habits affect anemia^2,21^. Further, taking perinatal or iron supplement is known to affect anemia status, however, no such data were available for the current study. In addition, when focusing on women of reproductive age and history of pregnancy and childbirth are highly relevant. Menstrual status, such as being pre-, peri-, and post- menopausal, is also essential to consider with regards to the presence of anemia. It has been found that the risk of anemia is higher in women who have menstrual disorders and menorrhagia and is reduced by the use of oral contraception^2,24^.

The main strength of this study was that this was the first investigation to have included age-based comparisons in the identification of factors related to anemia. Furthermore, the use of health check-up data meant that Hb data from about 10,000 adult women were available, and this study identified the actual anemia status of Japanese adult women. In addition, because there was a wide age distribution, and the median age was 42.0 years, there were many participants with a current medical history of gynecological diseases, as the incidence of these increases with age. It was, therefore, possible to detect a significant association between two gynecological diseases and anemia. In future research, factors related to anemia in women of different ages should be investigated using data in which the distribution of ages is uniform. Additionally, in future studies, lifestyle factors related to anemia should be clarified, taking into consideration nutritional intake and women’s menstruation status, including the menstrual cycle, irregular vaginal bleeding, subjective symptoms, and history of medical treatment.

## Conclusion

This study revealed factors related to anemia by age group. In women aged 35 and over, a current medical history of uterine myoma was associated with a higher risk of anemia, Women aged 35 and over who were overweight and reported a weight gain of 3 kg or more in the past year were found to be at lower risk of anemia. Women aged 35 and over who reported currently smoking and having smoked in the past were also found to be at lower risk of anemia. In both age groups, women who reported that they drank alcohol habitually were also found to be less likely to have anemia. It is important for women to have their Hb checked regularly. Factors related to anemia should consider including a woman’s age and lifestyle rather than generalizing the same factors to apply to all adult women.

## Methods

### Study design and participants

This study was a secondary data analysis using the St. Luke’s Health Check-up Database. Data from participants aged between 20 and 49 (i.e., of reproductive age) who had attended an annual health check-up at the Center for Preventive Medicine, St. Luke’s International Hospital in Tokyo, between April 2016 and March 2017 were selected. Personal information, such as medical record IDs, names, and addresses were not obtained. No pregnant women received this annual medical check-up at this facility. Questionnaire used in the medical health check-up indicated that pregnant women were considered to be ineligible to receive the medical check-up.

### Procedures

#### Annual health check-up

Approximately 40,000 people undergo an annual health check-up each year at the facility where the data were collected. This health check-up is composed of physical examinations to identify physical problems, and a questionnaire to collect information regarding a patient’s lifestyle habits and medical history. About 70% of examinees underwent their annual health check-up through their company’s employee health insurance.

### Hematologic data

The health check-up medical staff manually collected the venous blood using a blood collection tube to which the anticoagulant EDTA-2K was added. Hb concentration levels were measured using an Automated Hematology Analyzer (XN-1000, Sysmex, Kobe, Japan), and quality control was performed using a dedicated machine.

### Variables

#### Definition of anemia

In this study, anemia was defined as Hb <12.0 g/dL. The WHO classifies anemia status in non-pregnant women (above 15 years old) as mild (Hb in the range 11.0–11.9 g/dL), moderate (8.0–10.9 g/dL), or severe (<8.0 g/dL)^1^. Hb was considered biologically implausible when it fell outside the range 4.0–18.0 g/dL; these cases were treated as missing data^25^.

#### Socio-demographics and health behavior

Several independent variables related to socio-demographics characteristics and health behavior were considered. These were as follows: BMI, which was measured and calculated as kg/m^2^ during the annual health check-up and then categorized as less than 18.5 (underweight), 18.5–24.9 (normal weight), 25.0–29.9 (overweight), or 30.0 or more (obese); weight change over the past year (no change, 3 kg or more increase, or 3 kg or more decrease); smoking habits (non-smoker, current smoker, or smoked in the past); drinking habits (non-drinker, occasional drinker, or habitual drinker); sleeping habits (7 hours or more, or 6 hours or below of sleep); and exercise habits (no exercise, exercising 1–2 days per week, exercising 3–5 days per week, or exercising almost every day).

There were 6,269 women who answered the question about sleeping habits, and 4,329 who did not respond to this question. Some responses to this question clearly represented errors, as the number of hours of sleep reported ranged from 0 to 77. The mean ± SD was 6.1 ± 3.1 hours, and 99% of the respondents reported sleeping for 16 hours or less. Thus, in the present study, only data from the 6,207 women who reported sleeping between 1 and 16 hours were included in the analyses.

### Statistical analysis

The percentage of missing values for all variables was checked and missing data were excluded from each analysis. Normality of continuous variables was confirmed using the Kolmogorov-Smirnov test, and measures of skewness, and kurtosis. All variables were compared between the two groups of interest (anemia vs. without anemia) using the Student’s *t*-test, Welch’s *t*-test, or Mann-Whitney U test for continuous variables, and the Chi-squared test or Fisher’s exact test for categorical variables.

A multiple logistic regression analysis was performed to identify factors related to anemia while controlling for confounding factors. Variables that have been found to be related to anemia in previous studies and those with P values < 0.05 in the bivariate analysis were used for the multiple logistic regression model. Variables previously shown to be associated with anemia include age, BMI, and smoking status. Previous studies have pointed out a relationship between age and anemia, in that the risk of anemia is elevated in young people aged 15–24 and in those in their 40 s^25,26^, and have also identified an association between anemia and excessive obesity or underweight;^17,18^ additionally, the WHO emphasizes the fact that smoking increases Hb^1^. In guidelines published by the WHO^1^ and in previous studies^23^, it has been pointed out that smoking induces a rise in Hb. To adjust for this, following a method recommended in previous research^27^, another logistic regression analysis was attempted. We subtracted 0.3 g/dL from the measured Hb of those who reported in the questionnaire that they were current smokers. In the case of an absolute Spearman’s rank correlation coefficient between two independent variables being above 0.4 after bivariate analysis, only one of the two variables was included in the multiple logistic regression analysis owing to multicollinearity. Two-tailed P values < 0.05 were considered to indicate statistically significant comparisons. All analyses were performed using the SPSS software package (version 25.0) for Windows (IBM Corp., Armonk, NY, USA).

### Ethical considerations

Patients undergoing medical check-up were informed at their check-up that their medical data could be used in research conducted at universities and research institutions. If they did not wish to participate in such research, they submitted a non-participation in research statement and their data were excluded. Additionally, an overview of this research has been presented in advance in a document on the website of the facility where the study took place and of the Department of Midwifery and Women’s Health at the University of Tokyo. The study was approved by the Research Ethics Committee of St. Luke’s International University (No.18-A027), and the Graduate School of Medicine, the University of Tokyo (No.11977). The committees approved the form of opt-out consent for the participants in lieu of written informed consent. This study’s protocol complied with the principles of the Declaration of Helsinki^28^.

## Data Availability

The data that support the findings of this study are available from the Center for Preventive Medicine, St. Luke’s International Hospital, but restrictions apply to their availability. The data were used under license for this study, and are not publicly available. Data are, however, available from the authors upon reasonable request and with the permission of the Center for Preventive Medicine, St. Luke’s International Hospital.
